# How Food Choices Impact on Male Fertility

**DOI:** 10.1007/s13668-023-00503-x

**Published:** 2023-10-20

**Authors:** Giulia Pecora, Francesca Sciarra, Elena Gangitano, Mary Anna Venneri

**Affiliations:** Department of Experimental Medicine, Sapienza University of Rome, Viale Regina Elena 329, 00161 Rome, Italy

**Keywords:** Male infertility, Nutrition, Dietary patterns, Food choice, Semen quality, Sexual hormones

## Abstract

**Purpose of Review:**

Increasing evidence on the significance of nutrition in reproduction is emerging from both animal and human studies, suggesting an association between nutrition and male fertility. Here, we have highlighted the impact of the various food groups on reproductive hormones and on spermatogenesis, and the effects of classical and latest dietary patterns such as Mediterranean diet, Western diet, intermittent fasting, ketogenic diet, and vegan/vegetarian diet on male fertility.

**Recent Findings:**

Nutrients are the precursors of molecules involved in various body’s reactions; therefore, their balance is essential to ensure the correct regulation of different systems including the endocrine system. Hormones are strongly influenced by the nutritional status of the individual, and their alteration can lead to dysfunctions or diseases like infertility. In addition, nutrients affect sperm production and spermatogenesis, controlling sexual development, and maintaining secondary sexual characteristics and behaviors.

**Summary:**

The consumption of fruit, vegetables, fish, processed meats, dairy products, sugars, alcohol, and caffeine importantly impact on male fertility. Among dietary patterns, the Mediterranean diet and the Western diet are most strongly associated with the quality of semen. Nutrients, dietary patterns, and hormonal levels have an impact on male infertility. Therefore, understanding how these factors interact with each other is important for strategies to improve male fertility.

## Introduction

Nutritional status is directly related to eating habits, and adequate nutrition is fundamental for health. In recent years, unhealthy eating patterns, characterized by greater consumption of saturated fats, trans fatty acids, simple sugars and high sodium content, and lower consumption of foods rich in antioxidants, such as fruits and vegetables [1], have spread. Unhealthy eating patterns and a sedentary lifestyle contribute to the increased incidence of overweight and obesity, which are risk factors for many chronic diseases, such as cancer, diabetes, and cardiovascular disease and male infertility [2].

Physiologically gonadotropin-releasing hormone (GnRH) causes the pituitary gland in the brain to make and secrete the hormones luteinizing hormone (LH) and follicle-stimulating hormone (FSH). These hormones target the testis; in particular, LH acts on the Leydig cells, while FSH acts on the Sertoli cells. The Leydig cells are the primary source of testosterone (Te) and androgens which have a crucial role in fertility, including sperm production and spermatogenesis, controlling sexual development, and maintaining secondary sexual characteristics and behaviors [3–5].

**Fig. 1:**
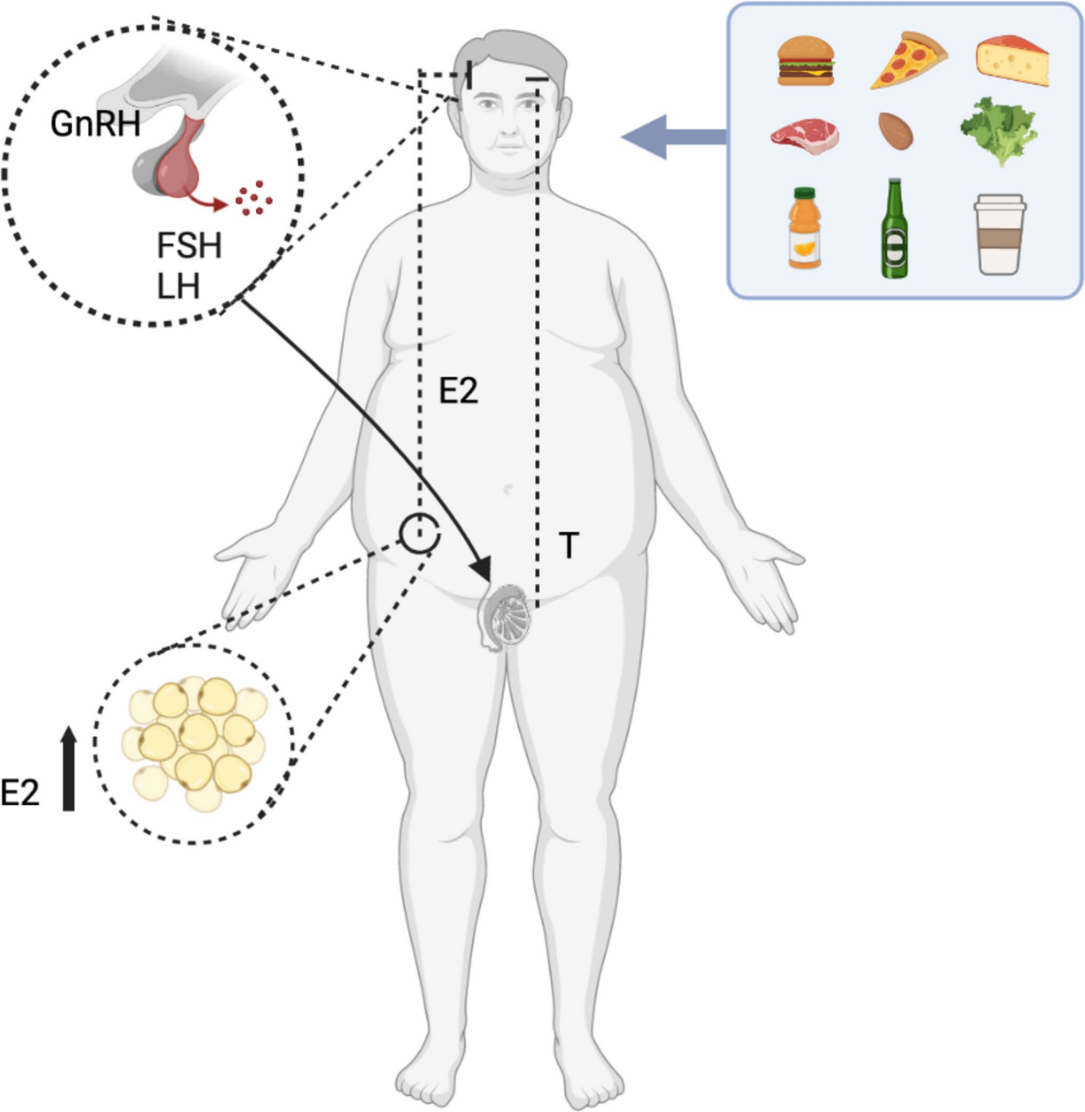
Gonadotropin-releasing hormone (GnRH) is secreted from the hypothalamus by GnRH-expressing neurons. The anterior portion of the pituitary gland produces luteinizing hormone (LH) and follicle-stimulating hormone (FSH), and the gonads produce estrogen (E2) and testosterone (Te). The increase in E2, in turn, acts with a negative feedback mechanism, on the hypothalamic-pituitary axis, with consequent inhibition of the release of GnRH and pituitary gonadotropins. These hormones target the testis; in particular, LH acts on the Leydig cells inducing the synthesis of steroid hormones, while FSH acts on the Sertoli cells, stimulating spermatogenesis. The increase of estrogens was related to intake of food pattern like red meat, dairy product, alcohol, SSB, sugars, and soy

In obesity, the increased adipose tissue determines an increase in adipocyte aromatase activity and a consequent increase in the circulating levels of 17 beta-estradiol (E2). The increase in E2, in turn, acts with a negative feedback mechanism on the hypothalamic-pituitary axis, with consequent inhibition of GnRH, LH, and FSH (Fig. 1) [6]. BMI was negatively correlated with inhibin B levels and with FSH. Increasing BMI in males is associated with decreased levels of serum Te, sex hormone binding globulin (SHBG), and inhibin B and increased free androgen index and E2 levels. Such reduced pituitary FSH stimulation could adversely affect Sertoli cell function, inhibin B, and sperm production, as well as Leydig cell testosterone production. A hypothesized mechanism for these changes involves the aromatase enzyme, capable of converting steroid precursors into estrogens. Increased amounts of adipose tissue would lead to increased conversion of Te to estrogen reported associated with obesity. On the contrary, there was no significant association of testicular volume with BMI but the lower concentrations of inhibin B in obese males seems to indicate decreased tubule function resulting in decreased tubular volume [2, 7].

In addition to these factors, an unbalanced diet leads to a low-grade systemic inflammatory state that can impact on spermatogenesis [8]. In this regard, men who are overweight and suffer from metabolic syndrome may be at higher risk of infertility due to abnormal hormonal regulation and radical oxygen species (ROS) production. Thus, an alteration of sexual hormones, obesity incidence, and inflammatory state can have a direct effect on Leydig cell dysfunction and poor semen parameters as the sperm concentration, motility, vitality, and morphology [9].

Since the eighties, the excessive or insufficient intake of some substances, as trans fatty acids and zinc respectively, has been considered a determining factor for sperm function, fertility, and the functionality of the reproductive system [10, 11].

**Fig. 2:**
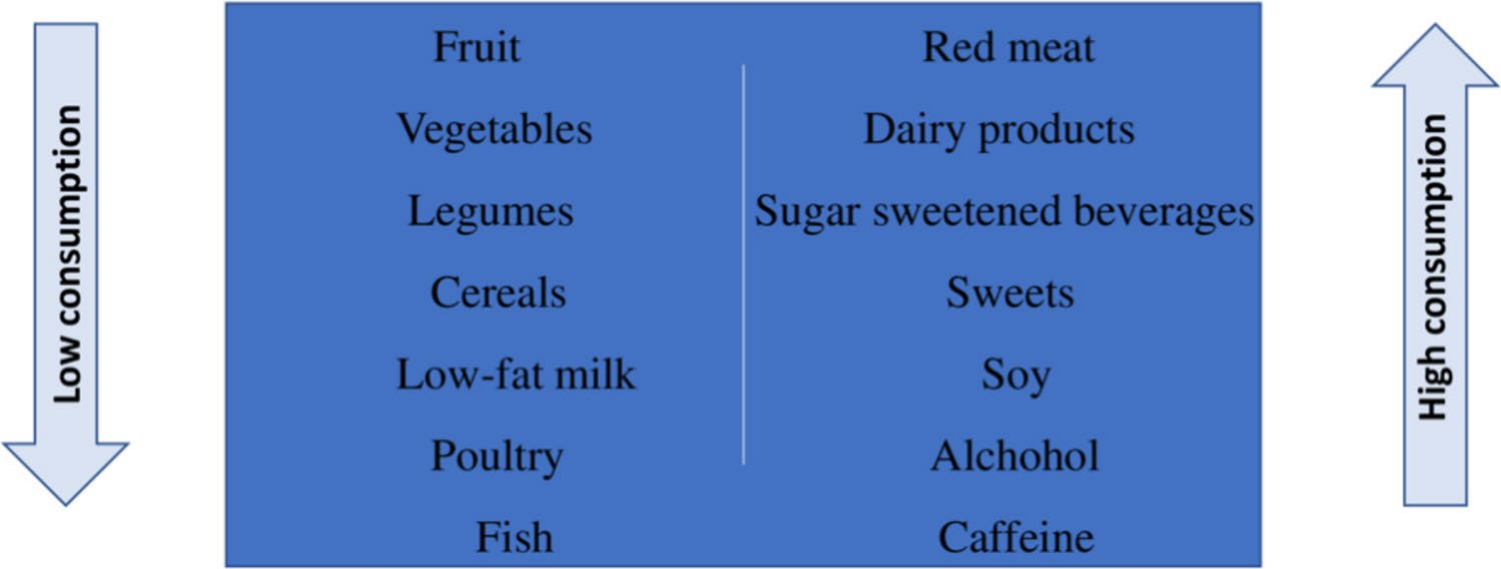
Eating habits associated with male infertility

Recently, several studies highlighted how diets rich in fish, shellfish and seafood, poultry, cereals, vegetables and fruit, and dairy products (low fat content) are positively correlated with semen quality [12, 13]. Conversely, diets rich in processed meats, soy, potatoes, full-fat dairy products, coffee, alcohol, sugary drinks, and sweets seem to worsen semen quality [12, 14] (Fig. 2).

The father’s nutritional status during the periconceptional period is particularly important for the efficient fetal development and the growth of the progeny [15, 16]. In addition to age and environmental exposures, food may also be a risk factor for de novo mutations in fathers with possibly transgenerational consequences. All these events are adaptive and may change during life conferring, or not, predisposition to chronic disease and cancer [15, 16].

The aim of this review is to summarize the available findings on food and dietary patterns and their impact on male fertility.

## Methods

We conducted a narrative review of the literature to evaluate the relationship between food groups, dietary patterns, and male fertility. We searched the Medline (PubMed) database between 2010 and January 2023 using the following search terms: male fertility OR fertility OR male infertility OR reproduction OR semen quality OR sperm OR asthenozoospermia OR teratozoospermia OR sperm DNA damage OR oligozoospermia OR oligoasthenozoospermia OR oligoasthenoteratozoospermia AND nutrition OR nutrient OR diet OR food OR vitamins OR antioxidants OR cereals OR meat OR fatty acids OR poultry OR vegetables OR fruit OR nuts OR fish OR shellfish OR sweets OR sugars OR sugar-sweetened beverages OR legumes OR milk OR cheese OR dairy products OR eggs OR caffeine OR alcohol OR ethanol OR sugar OR consumption OR soy OR estrogens OR Mediterranean diet OR Western diet OR Vegetarian diet OR Vegan diet OR Ketogenic diet OR Intermittent fasting.

Additional studies were identified from the study reference lists. We included case–control, cross-sectional and observational prospective and retrospective studies, review articles, meta-analysis, RCTs, and animal studies.

## Food Groups and Their Influence on Fertility

### Dairy Products and Meats

Saturated fatty acids (SFAs) and trans fatty acids (TFAs) are present in most foods of animal origin including meat, dairy products, and hydrogenated vegetable oils and have a role in the increasing incidence of obesity, coronary heart disease, diabetes, cancer, and also infertility [17]. In this regard, several studies have identified a negative correlation between a diet rich in trans fatty acids and sperm concentration, ejaculate volume, motility, better morphology [18–20], and high Te level [21].

The impact of SFAs in the deterioration of seminal quality could be attributed to their ability to induce a low-grade state of inflammation and the increased concentration of cholesterol in the membrane of spermatozoa, damaging their structure and compromising the quality of the gamete. Furthermore, the mitochondria, following the increase and accumulation of substrates, may not be able to oxidize all the lipids that accumulate in them, thus triggering the process that leads to an excess of radical oxygen species (ROS) production by sperm [22], and an adverse impact on motility and DNA integrity (Table 1) [23]. On the contrary, the polyunsaturated fatty acids (PUFAs), particularly the omega 3 PUFAs, have a positive effect on seminal pH, semen volume, sperm motility, sperm concentration, and vitality [24].

**Table 1:** The impact of food groups on male fertility markers

**Food groups**			**Markers of male fertility**									
		**Concentration**	**Vitality**	**Volume**	**Progressive motility**	**Normal forms**	**ROS production**	**DNA integrity**	**Te levels**	**FSH levels**	**LH levels**	**E2 levels**
**Meat**		↓ [18, 19, 152]			↓ [25••, 152]	↓ [31, 152]						
**Dairy products**	High-fat products	↓ [30]			↓ [29–31]	↓ [29]				↑ [29]		
	Low-fat milk	↑			↑ [31]		↓ [32]					
**Fruit, vegetable, legumes and cereals**		↑ [27, 41••, 47, 49, 50]	↑ [41••, 47]		↑ [27, 31, 40, 41••, 47, 49, 50, 124]	↑ [27, 40, 47, 56]	↓ [41••, 45, 49, 50, 153]	↑ [50]	↑ [48, 49]	↑ [48]	↑ [48]	↑ [48]
	Dried fruit	↑ [52, 54]	↑ [51–53]		↑ [51–53]	↑ [51–53, 55]	↓ [52, 154]		↑ [52]			
	Soy	↓ [59, 136, 155]			↓ [59, 136]	↓ [59]	↑ [59]			↓ [132, 134]	↓ [132, 134]	↓ [132]
**Fish and shellfish**		↓ [70]			↑ [31, 40, 124, 128]	↑ [31, 40, 70]						
**Sugars and sweet beverages**		↓ [40, 79, 80, 128]		↓ [79]	↓ [31, 40, 78, 79]	↑ [81•]	↑ [78]	↓ [78]		↑ [80]		↑ [81•]
**Caffeine**		↓ [84, 87, 92]	↑ [89, 92]		↑ [83, 89, 91•, 92]	↓ [83, 84, 87, 90, 95]		↓ [87]	↑ [84]			
**Alcohol**		↓ [96, 97, 102, 103]		↓ [96, 97, 101–103]	↓ [96, 97, 102]	↓ [96, 97, 102, 103]	↑ [99, 100, 105]	↓ [102]	↓ [96]	↑ [96]	↑ [96]	↑ [96]

Notably, the references in Table 1 are mostly observational studies. Therefore, there is a need for RCT studies to support clinical application

↑ indicates an increase of fertility marker; ↓ indicates a reduction of fertility marker

Regarding meat consumption, the intake of total meat, unprocessed meat, and raw vegetables may reduce the risk of asthenozoospermia, while a higher consumption of processed meat (about 2 or more servings/day) may increase the risk [25••]. Poultry intake was linked to a higher fertilization rate; conversely, processed meat consumption was associated with a lower fertilization rate in couples undergoing assisted reproduction [26]. Likewise, in the study of Braga et al., red meat consumption was inversely related to implantation and pregnancy rate in couples undergoing intracytoplasmic sperm injection (ICSI) [27].

To note, the greatest amount of meat consumed in the western countries derives from intensive farming and contains high concentrations of xenoestrogens and in some cases steroids, which, being endowed with estrogen-like actions, can become responsible for a decrease in male fertility impacting on semen quality and reproductive hormone levels [23, 25••, 26]. Among xenoestrogens, polychlorinated biphenyls (PCBs) were detected in the seminal plasma of infertile men and the concentration of phthalate esters (PEs) was significantly higher in infertile men compared with controls. In particular, in infertile men, the xenoestrogen concentrations were inversely proportional to the total motile sperm counts [28].

Analyzing the dietary habits of patients affected by oligoasthenoteratozoospermia (OAT) was found that the intake of dairy fat was positively associated with the risk of OAT [30]. Conversely, the use of low-fat milk was associated with a lower risk of asthenozoospermia (Table 1) [31]. In addition, some studies evaluated the relationship between the intake of dairy products and male fertility, and observed that the intake of dairy products was inversely related to sperm morphology and progressive motility, but positively correlated with FSH levels (Table 1) [29].

Other important evidence comes from the recent work of He et al., in which it was shown that lactoferrin and milk attenuate the dysfunctions of spermatogenesis, improving disordered autophagy, apoptosis, and oxidative stress, in the male mice reproductive system [32].

As for meat, cow’s milk has also been suggested as a possible source of xenoestrogens that could affect human health, even if the amounts of estrogens in cow’s milk are usually too low to affect humans [33]. The presence of environmental contaminants in dairy products, such as pesticides and chlorinated pollutants [34], have been associated with lower sperm quality [28, 35] and elevated FSH levels and no alteration of other sexual hormones [36]. The data was interesting and is in contrast with the physiological effect of FSH on spermatogenesis in which the administration of FSH to normogonadotropic patients with male factor infertility induced a marked increase in sperm count, a slight increase in sperm motility [37]. The potential effects of fermented milk products on male reproductive health were investigated in animal models [38] identifying a protective effect of fermented goat’s milk on genomic stability, oxidative stress, and inflammation in rat testis during anemia recovery. In particular, the study of Hammami et al. on mice showed how kefir consumption recovers impaired spermatogenesis due to a high-fat diet and restores testicular barrier proteins [39].

### Fruits, Vegetables, and Legumes

Fruit and vegetables are the main sources of antioxidant substances in nature. In a healthy and balanced diet, the intake of these foods corresponds to 3 portions of fruit and 2 portion of vegetables per day [40].

Fruits and vegetables have a low-calorie content and provide water, simple sugars (fructose), vitamins, minerals (potassium and magnesium), fibers, and phytochemicals. Phytochemicals (organic acids, polyphenols, and oligosaccharides) are organic compounds of vegetable origin without nutritional properties, with antioxidant action against free radicals and polyunsaturated fatty acids and contribute to the development of intestinal bacterial flora [40]. Antioxidant power of vitamins has been associated with the ability of sperm to produce ROS [41••]. Several studies have confirmed the negative effects of ROS accumulation on seminal quality, chromatin integrity, and on the rate of abortion and malformations in offspring [8, 42]. Interestingly, some human studies have shown that antioxidant supplementation can reduce the oxidative stress in spermatozoa [43, 44].

In this regard, some studies have highlighted the protective role of some fruit juices and extracts on male fertility. In particular, the antioxidant activity of vitamins and phytochemicals, contained in pineapple, tomato, strawberry, elderberry, pomegranate, cherry laurel, grape, raspberry, and white mulberry, can have a protective role in testicular damage rodent models. Altogether, the main effects would be to restore serum Te levels, reduce testicular oxidative imbalance, improve the morphological condition of the seminiferous tubules, and thus improve seminal quality including concentration, motility, and morphology [45–49].

Moreover, resveratrol, a natural non-flavonoid polyphenol widely present in grapes, peanuts, berries, and red wine, has a positive effect on sperm motility at low concentrations, whereas at higher concentrations, it has a detrimental effect on sperm motility and has a protective role against sperm DNA damage caused by oxidative stress [50].

Some human studies have shown the protective effect of the consumption of dried fruit, in particular walnuts, date palm, hazelnut, and chestnut on male fertility [12, 51, 52]. The consumption of walnuts daily added to a Western-style diet improved effects on sperm vitality, motility, and morphology and had an impact on sperm DNA methylation (Table 1) [53], testicular antioxidant function, and semen quality. The consumption of chestnut polysaccharides has an impact on restoring spermatogenesis [54]. In fact, the presence of omega-3 alpha-linolenic acid (ALA) is crucial for some cellular functions such as phagocytosis of residual bodies by Sertoli cells, morphology, and fluidity of the sperm membrane [55].

Beyond vitamins, folates, mainly present in green leafy vegetables, protect the DNA from damage, by reducing the concentration of homocysteine through its re-methylation into methionine. In fact, homocysteine, functioning as a strong oxidative stress factor, can increase the reactive oxygen species which in turn can compromise the concentration of spermatozoa and their mobility. Furthermore, folates play a central role in spermatogenesis with a significant increase in total normal sperm count and a minor increase of abnormal spermatozoa in double-blind, randomized, placebo-controlled trial sub-fertile and fertile men (Table 1) [56].

The supplementation of folate, zinc, and antioxidants (vitamin C, vitamin E, and beta-carotene) in men with adequate nutrition is associated with lower frequencies of sperm with aneuploidy [57].

Furthermore, fruits, vegetables, legumes, and whole grains are the main source of fiber. It was highlighted that the consumption of fibers reduces plasma estrogen levels, due to their direct link with non-conjugated estrogens of food origin, limiting their intestinal absorption [58].

Among legumes, soy, in contrast to the other legumes, may have deleterious effects on spermatogenesis, even if the results are not concordant across the studies. This negative influence may be due to the presence of phytoestrogens [59, 60]; in fact, the intake of soy foods and sources of isoflavones is inversely related to the concentration of sperm [58] and free Te levels [61, 62]. Some studies have shown a deleterious impact of soy consumption on neonatal exposure male rats or humans on reproductive defects [63–66] and decreased weight or size of testicles [67]. Instead, other studies have not shown effects of soy on the concentration of gonadotropins and sex hormones, or on seminal quality [61, 68] while other identified lower levels of FSH [67] and Te [69] (Table 1).

### Fish and Shellfish

Fish consumption is associated with a higher number of total spermatozoa and a higher percentage of morphologically normal spermatozoa (Table 1) [70]. Specifically, dark meat, such as salmon and tuna, is associated with an increase in the total number of spermatozoa while the consumption of fish with light flesh, such as cod and halibut, correlates with a greater expression of typical forms of sperm [20].

The potential benefits deriving from fish and shellfish can be related to their content in omega-3 fatty acids or PUFAs, such as docosahexaenoic acid (DHA) and eicosapentaenoic acid (EPA). Omega-3 essential fatty acids are important components of sperm cell membranes [71, 72], and omega-3 supplementation in a double-blind, placebo-controlled, randomized study conducted in subjects affected by idiopathic oligoasthenoteratospermia resulted in an improved the sperm cell total count, and both SOD-like and catalase-like activity [73].

Despite the beneficial effect linked to PUFAs, fish can compromise fertility because of toxic molecules. A Swedish study reported that organochlorines, pollutants present especially in mollusks and seafood, are associated to the alteration of seminal parameters such as total motility and chromatin integrity [74]. Heavy metals as mercury (Hg), found in high concentrations in the adipose tissue of marine fauna, affect spermatogenesis [75]. Furthermore, a recent study showed that the consumption of canned fish was inversely related to sperm immobility, while a high consumption of fresh fish increased the percentage of immotile sperm in Iranian infertile men [76]. However, the authors should be considered that in Iranian habits, fresh fish is eaten fried and therefore seems to have a negative effect on seminal quality.

Taking all these considerations, further investigations are needed to confirm the relation between fish and male infertility.

### Sugars and Sugar-Sweetened Beverages

Glucose metabolism is essential for spermatogenesis. Glucose enters the spermatozoa by facilitated diffusion, through the expression of glucose transporters (GLUT) on the cell membrane. However, an excessive intake of glucose due to diets rich in sugars, sweets, potatoes, and foods with a high glycemic index influence semen quality [77] because hyperglycemia affects sperm motility and sperm maturation [78]. Sugar-sweetened beverage (SSBs) intake was inversely correlated with low semen quality in terms of total sperm count, semen volume, and motility (Table 1) [79]. The consumption of SSBs is also associated with a lower serum inhibin-B/FSH ratio [80] and higher E2 level that are correlated with low sperm production and quality (Table 1) [81•]. Hatch et al. observed that SSBs, particularly sodas and energy drinks, were associated with lower fecundability in a North American preconception cohort [82].

Moreover, the consumption of sweets and sugary drinks is associated with a higher incidence of obesity, type 2 diabetes mellitus, and metabolic syndrome, all conditions in which insulin resistance occurs, which leads to an increase in oxidative stress [78].

### Caffeine and Alcohol

Among the various eating habits, the increasingly frequent intake of coffee has been associated with altered spermatogenesis [83], and with increased blood Te levels that leads to a decrease in circulating gonadotropins (Table 1) [84]. However, the association between caffeine and fertility rates is not defined [85]. Recently, Ricci et al. have shown that a moderate caffeine intake by women and men in the year prior to the assisted reproductive technology (ART) procedure was not associated with negative outcomes [86].

The relationship between caffeine and fertility remains unclear. In fact, if on the one hand it seems to correlate with a decrease in the total number of spermatozoa [84, 87] and with an increased number of atypia and sperm DNA damage (Table 1) [87], on the other hand, it seems to improve the energy metabolism of Sertoli cells [88] and sperm motility [89, 90, 91•, 92]. Akomolafe et al. showed that caffeine reversed the alteration of fertility parameters in a rat model through the modulation of the steroidogenic enzymes, improving sperm quality and attenuating the oxidative damage in testicular and epididymal tissues [92]. In rodents, caffeine exposure has shown an effect on the weight and size of reproductive organs [93], on the testicular microarchitecture, and on the germ cell proliferation [94].

It has also been highlighted that the risk of dyspermia is directly proportional to the number of cups of coffee consumed daily [83, 84, 95]. In contrast, a study conducted on the Danish population found only a reduction in the total number of spermatozoa in relation to excessive caffeine intake [87].

Several animal and human studies have focused on the impact of ethanol (EtOH) consumption on reproductive hormonal regulation, semen quality, gene transcription, genetics, and epigenetics regulation [96, 97]. Moderate alcohol consumption does not seem to have a major effect on the seminal fluid parameters [98]; on the contrary, its abuse has a negative effect on the entire male reproductive system, through various pathogenic mechanisms. In fact, EtOH and its metabolites, by influencing the hypothalamic pituitary axis, induce a suppression of gonadotropin production, causing a decrease in Te levels and an altered relationship between free Te and free E2 [96]. Moreover, alcohol has a direct negative impact on spermatogenesis (Table 1) [96] as it contributes significantly to the increase of oxidative stress and the formation of ROS [99, 100]. Alcohol may also alter the secretion of the accessory glands, resulting in a decreased seminal quality [101] in terms of volume, concentration, morphology, motility, epigenetic regulation, expression of genes, and protein involved in sperm functions, DNA integrity, and chromatin condensation [102]. These alterations can occur both in the chronic alcoholic and in the occasional drinker, causing a reduction in seminal volume, a reduction in the total number of spermatozoa, and an increase in atypia (Table 1) [97, 103].

Only a few studies showed how moderate alcohol consumption is related to male fertility. Ricci’s human study associated moderate alcohol consumption to an improvement of seminal quality in terms of volume and concentration [98]. Instead, the sub-chronic ingestion of alcohol negatively affects sperm morphology, capacitation parameters, and IVF dynamics on mice. Interestingly, two animal studies have focused on understanding the role of alcohol in the relationship between gut and male fertility. In particular, the work of Li et al. showed that chronic alcohol consumption induces intestinal dysbiosis, which leads to testicular inflammation and impaired sperm quality [104]. In the same way, Aderara et al. sustained that the exacerbating effects of ethanol on ulcerative colitis-induced testicular dysfunction are related to increased oxidative stress and inflammation [105].

Rao et al. conducted a systematic review and a dose-response meta-analysis to evaluate the association between caffeine and alcohol consumption and in vitro fertilization (IVF) and ICSI outcome. The authors reported that men’s weekly alcohol consumption greater than 84 g was associated with decreased live birth rate after IVF/ICSI treatment [106]. In contrast, Karmon et al. reported that the intake of alcohol was directly associated with live birth after assisted reproductive technologies, but not with semen parameters [107]. Instead, caffeine intake seems to be negatively associated with ICSI [107].

## An Overview of Dietary Patterns

### Mediterranean and Western Diet

Over the last few years, nutritional epidemiology has shown that an adequate caloric intake, the reduced consumption of red meat in favor of white meat, the increased intake of fruit and vegetables, and the replacement of saturated fats with mono fats and polyunsaturated fats benefit parameters of human health, including fertility [108–110]. A greater compliance to the Mediterranean Diet (MedDiet) is associated with better semen quality parameters as sperm concentration, total sperm count, total, and progressive motility [111, 112] but not with semen volume [13], in normal subjects, and among men of subfertile couples attempting fertility [113].

Montano et al. studied healthy young men living in highly polluted areas of Italy in a randomized trial, and reported that a lifestyle intervention based on MedDiet and physical activity resulted in an increase of sperm concentration, total and progressive motility, increased percentage of spermatozoa with normal morphology, and a decrease of round cell concentration [114••].

Additionally, other studies have investigated the impact of the MedDiet on male infertility by comparing it to other types of diets including Western diet. The Western diet is characterized by high intakes of pre-packaged foods, refined grains, red meat, processed meat, high-sugar drinks, candy and sweets, and fried foods [115]. In vitro and in vivo studies in rodents have elucidated the consequences of chronic intake of high-fat foods on male fertility. The high-fat diet (HFD) can cause high expression of heat shock protein 60 (HSP60) in spermatozoa triggering apoptosis [116], altered DNA methyltransferase enzymes and global methylation in gonads and testes [117, 118], decreased sperm quality by disrupting energy metabolism in Sertoli cells [119], and aberrations in the testicular proteome [120].

Cutillas-Tolin et al., in their study, showed that the Mediterranean pattern is positively associated with total sperm count. Instead, the Western pattern is positively related to the percentage of morphologically normal sperm and inversely related to sperm concentration among overweight or obese men but not among lean men [121]. The effects of the Mediterranean and Western diet on testicular function showed adverse consequences of high-saturated fatty acids and cholesterol diets on testicular function, supporting benefits of a Mediterranean diet and virgin olive oil to improve male fertility [122].

Yörüsün et al. confirmed that SSB, red meat, and organ meat consumption negatively correlate with sperm parameters. On the other hand, the consumption of fish, egg, and nuts is positively correlated with sperm parameters [123]. Men with higher adherence to a “prudent” or “health-conscious” pattern, a diet rich in fruit, vegetables, whole grains, legumes, nuts, fish, and low-fat dairy products, bearing close resemblance to the Mediterranean pattern, were associated with higher sperm concentration, progressive motility [124], and total sperm [125, 126] and had an inverse association with the odds of infertility [127]. Furthermore, health conscious dietary pattern, which comprises high intakes of fruit, vegetables, fish and other seafood, whole grains and legumes and low intakes of fatty sauces, meat products, refined grains, sugar, and confectionary, was associated with lower sperm DNA damage among subfertile men of couples undergoing IVF/ICSI in the Netherlands [128]. Recently, Muffone et al. conducted a systematic review with meta-analysis to understand whether high MedDiet adherence could be a contributing factor to positive fertility outcomes in infertile men and women. According to this work, the current evidence of high adherence to MedDiet and fertility markers is insufficient to support their clinical application, even though it indicates sperm improvement and a possibility of better pregnancy outcomes [129].

### Vegetarian and Vegan Diet

Individuals who follow a vegetarian diet abstain from the consumption of animal meat for cultural, environmental, economic, health, political, or religious reasons. There are different variations of the vegetarian diet such as ovo vegetarians who consume eggs but no dairy products, and lacto-ovo vegetarians who include both milk and eggs in their diet [130]. Conversely, vegans consume only plant products. Generally, most vegetarians replace meat products with soy in their diet to ensure protein intake. A vegetarian diet rich in soy foods has been recognized as healthy for decades, due to the positive effects on metabolic parameters and cardiovascular risk [131] but actually following the identification of isoflavones in soy foods, which exert estrogen-like effects, have been shown to have a negative role on fertility [132–136]. Orzylowska showed that there is a reduction in sperm concentration and motility but no alterations in sperm morphology and sperm chromatin integrity in vegetarians [136]. While in vegans an inefficient sperm hyperactivation was highlighted [136], as well as higher sperm DNA methylation levels in metabolism-related genes [137] and alterated spermatozoa fatty acids composition [138], results obtained in Kljajic’s study supported the favorable effect of a vegan diet on semen parameters as total sperm count, percentage of progressive motility, DNA integrity, and oxidation–reduction potential [139]. Of note, evaluations on the effects of vegetarian diets on male fertility should be integrated, taking into consideration the eventual exposure to pesticides [140], mycotoxins [141], and the eventual shortage of zinc and vitamin B12 [142].

### Intermittent Fasting Diet

Intermittent fasting is an increasingly popular strategy for weight loss, but concerns have been raised regarding the effects of fasting on the reproductive health of women and men. Intermittent fasting reduced Te levels and the reductions did not appear to be related to the duration of intervention [143].

Sex hormone binding globulin (SHBG) is the major serum carrier of sex hormones. The reduction of Te lead to intermittent fasting does not impact on SHBG levels [144, 145] probably because the Te became bound to other carriers such as albumin. Low Te levels can negatively affect metabolic health, muscle mass synthesis, and libido in males [143]. However, the reduction of Te impacts on the reduction of fat mass but not to fat-free mass [144, 145]. There is no data in the literature on the impact of intermittent fasting on seminal fluid.

### Ketogenic Diet

Ketogenic diet is characterized by a metabolic switch that determines the development of ketosis, as fat is used as a primary source of energy, instead of carbohydrates. The oxidation of the fatty acids determines the production of ketone bodies that are used as fuel by many tissues, including the central nervous system, skeletal muscle, and the heart. It has been developed as an adjuvant therapy in refractory epilepsy in children, and nowadays, it is recommended in severe obesity. Ketogenic diet has also been proposed as an adjuvant therapy for other pathologies, as headache and COVID-19 [146–148]. There are few studies advocating the effects of a ketogenic diet on improving fertility, and most have focused on female fertility [62, 149].

In vitro study has demonstrated that ketone bodies are being utilized as an energy source for sperm movement. In an animal study, the ketogenic diet restored the sperm motility, the percentage of sperm with a normal morphology, and spermatogenic cell maturation in HFC-fed mice, even though there was no greater enhancement in Te levels [149]. Omowumi et al. investigated the effects of ketogenic diet in monosodium glutamate (MSG)–induced rat testicular toxicity [150]. MSG is a flavor enhancing food additive found and is a major component of many proteins such as milk, meat, fish, and some vegetables. MSG treatment can cause an alteration of redox status, a reduction of testicular glycogen, a decreasing of NO level, and alteration of lipid profiling. This study showed that ketogenic diet improved the rat bio-chemical parameters as well as the testicular functional indices included testicular: alkaline phosphatase (ALP), acid phosphatase (ACP), cholesterol, protein, glycogen, and testicular lipid profile [150].

In addition, a ketogenic diet with curcumin supplementation could ameliorate Te levels, poor spermatogenesis, sperm parameters as motility and morphology, and reverse oxidative stress; inhibit inflammation; and inhibit apoptosis in the testes of low-carbohydrate-diet-fed mice [151].

## Conclusion

Several scientific studies highlighted that unbalanced diet could affect male fertility. Processed meats, dairy products, canned fish, sweets and sugary beverages, soy, and the abuse of alcohol and caffeine have a negative impact on semen quality. Instead, the consumption of fish, fruit, dried fruit, vegetables, legumes, cereals, and low-fat milk benefit male fertility. Among dietary patterns, the Mediterranean diet is positively associated with semen quality, while the Western diet negatively affects spermatogenesis. In the vegetarian diet, results are not clear, but the wide use of soy correlates with deleterious effects on spermatogenesis, probably because of its content of phytoestrogens. Some preliminary results suggest that the ketogenic diet may have a positive impact on spermatogenesis and increase the Te levels in men.

Despite the increasing amount of literature regarding the relationship between male fertility and diet, the current evidence of nutrition and fertility markers is insufficient to support their clinical application. Future research should also consider the need for randomized controlled trials.

Nevertheless, the work-up of the infertile patients should take into consideration also the nutritional aspect, to adjuvate medical therapies and personalize the medical approach. Thus, there is a need for more standardized integration of nutrition counseling into treatment delivery for infertility.

More research is warranted to further elucidate the complex mechanisms between diet and fertility outcomes, and the implications for public health and clinical practice.
